# Intravenous tranexamic acid significantly improved visualization and shortened the operation time in microscopic middle ear surgery: a randomized controlled trial

**DOI:** 10.1097/JS9.0000000000001366

**Published:** 2024-03-21

**Authors:** Yunpeng Zhang, Lin Han, Weisi Ding, Lan Gao, Yi Feng, Haiyan An

**Affiliations:** a Department of Anesthesiology, People’s Hospital, Peking University; b Department of Otorhinolaryngology, Head and Neck Surgery, People’s Hospital, Peking University, Beijing, People’s Republic of China

**Keywords:** microscopic, middle ear surgery, operation time, surgeon satisfaction, tranexamic acid, visualization

## Abstract

**Background:**

The microscopic middle ear surgery involves a limited operating space and numerous important anatomical structures in which good visualization is crucial, as even a small amount of bleeding can greatly affect the clarity of surgical field. This study aims to investigate whether intravenous 1 g of tranexamic acid can improve surgical visualization and further shorten the operation time in microscopic middle ear surgery.

**Methods:**

This study is a prospective, randomized, double-blind, controlled trial conducted from December 2021 to December 2022, enrolling patients who were scheduled for microscopic modified radical mastoidectomy due to chronic otitis media. In addition to standard techniques to optimize the surgical field, participants were randomized into the TXA (tranexamic acid) group (1 g diluted to 20 ml normal saline) and the control group (20 ml normal saline). The primary outcome was assessed based on the clarity of the surgical field using the Modena Bleeding Score. Secondary outcomes included operation time, the surgeon satisfaction with the visual clarity, postoperative 24 h coagulation parameters, and the incidence of adverse events. Student’s *t*-test, *χ*^2^ test, and ANOVA of repeated measures were used for statistical analyses.

**Results:**

A total of 28 patients were enrolled in each group using a 1:1 randomized allocation with similar demographic characteristics, including 24 male and 32 female individuals, and the mean age is 45.6±11.9 years. The surgical visualization in the TXA group was significantly better than that of the control group (2.29±0.46 vs. 2.89±0.31, *P*<0.001) as assessed by the Modena Bleeding Score. Furthermore, the TXA group demonstrated a shorter operation time compared to the control group (88.61±10.9 vs. 105.2±15.9, *P*<0.001) and higher surgeon satisfaction with surgical field (7.82±0.55 vs. 6.50±0.64, *P*<0.001). No statistically significant differences were found in postoperative coagulation parameters in the two groups. No TXA-related adverse events or complications occurred during the 12-month follow-up.

**Conclusion:**

Intravenous 1 g of TXA can further significantly improve the visual clarity in the microscopic middle ear surgery and shorten the operation time based on other standard measures implemented.

## Introduction

Modified radical mastoidectomy represents the most common surgical procedure in the middle ear for chronic suppurative otitis media in current practice, which is performed with the aid of a microscope or otoendoscope^1^. One of the major concerns during surgery is surgical field visualization, as clear visibility is crucial for recognizing important anatomical structures that contribute to surgical progression and patient safety. Impaired visualization can result in complications or prolonged surgical procedures. The surgical field of view is closely associated with bleeding because even small areas of bleeding can reduce visibility. A multitude of techniques are employed to reduce intraoperative bleeding and improve the clarity of the surgical field of view, such as deliberate hypotension and raising the head of the bed during surgery, topical vasoconstrictors, and total intravenous anesthesia^2^. Nevertheless, the above-mentioned methods are still not at a satisfactory level and will affect systemic hemodynamic stability and pose the potential risk of affecting vital organ perfusion, especially among vulnerable elderly patients with cardiovascular disease^3^.

TXA is a synthetic derivative of the amino acid lysine that acts by reversibly blocking lysine‐binding sites on plasminogen, which competitively prevents plasmin and lysine residues on the fibrin polymer from interacting, subsequent fibrin degradation is slowed, and exhibits an antifibrinolytic effect^4^. Intravenous TXA can rapidly exert antifibrinolytic effects with a half-life of about 2–3 h, and its reported adverse reactions are relatively mild, most commonly reported gastrointestinal events, such as nausea, diarrhea, etc.^5,6^. Although theoretically, TXA may increase the risk of thrombosis, the current meta-analysis results show that it does not increase the risk of vascular occlusive events and other complications^7^. Studies have shown that the plasma concentration of 5–10 ug/ml can quickly play an effective antifibrinolysis effect, and intravenous injection of 1 g TXA can maintain therapeutic plasma concentrations for more than 5–6 h^8^.

As a highly effective antifibrinolytic agent, there is strong evidence that TXA reduces blood loss in orthopedics, cardiac, trauma, and other major surgeries with a large amount of blood loss^9,10^. However, its application in microsurgery with less bleeding where high visual clarity is required has attracted less attention. Considering its highly effective antifibrinolysis properties, it is reasonable to speculate that TXA can provide a clearer surgical field of view and satisfactory operating conditions in microscopic middle ear surgery. Given this, this study will assess the effect of TXA during the microscopic modified radical mastoidectomy under the premise that standard techniques were applied to minimize blood loss. We hypothesized that intravenous TXA of 1 g would improve the clarity of the surgical field and may further shorten the operation time.

## Materials and methods

### Study design and participants

We conducted a prospective randomized, double-blind, controlled trial to explore whether intravenous TXA of 1 g before skin incision can further improve the clarity of the surgical field during microscopic modified radical mastoidectomy with all other measures implemented to improve the surgical visualization. Ethical approval was given by the Peking University People’s Hospital Research Ethics Committee (2021PHB173-001) and this study was registered on the Chinese Clinical Trial Registry (ChiCTR2100049183). Guidelines from the Helsinki Declaration were followed and reporting in this randomized clinical study adhered to the Consolidated Standards of Reporting Trials (CONSORT)^11^.

All patients aged 18–65 who were scheduled for microscopic modified radical mastoidectomy for chronic suppurative otitis media were eligible for inclusion in this study. Written informed consent was obtained from all recruited participants one day prior to surgery. Exclusion criteria included: allergy to TXA, ASA physical status III or greater, patients with severe liver or kidney disease, cardio-cerebrovascular problems, thromboembolic disorders, hypertension, seizures, coagulation dysfunction, use of anticoagulants, colored vision, pregnancy and lactating, lack of patient consent, and any additional surgical procedures to be performed simultaneously.

### Randomization and blinding

Eligible participants were randomly assigned to receive TXA or normal saline. Randomization was conducted using a computer-generated random number table, with an equal allocation ratio of 1:1, and the allocation group was stored within sequentially numbered, opaque, sealed envelopes, which were supervised by an investigator who was blinded of the group allocations, follow-up, or data analysis^12^. Before participants entered the operating room, anesthesiologists were given a syringe containing a total volume of 20 ml of either 1 g TXA or normal saline. Both participants and the medical staff (anesthesiologists and surgeons) were unaware of the randomization assignments. Premature unblinding was allowed only if necessary for patient safety.

### Anesthesia and surgery protocols

Upon arrival in the operating theater, standard monitoring by intermittent noninvasive blood pressure (NIBP), continuous ECG, and saturation with pulse oximetry (SpO_2_) was monitored continuously, and recorded manually at baseline and 10 min intervals. All patients received standardized general anesthesia using propofol/sufentanil/rocuronium for anesthesia induction and propofol/remifentanil continuous infusion for maintenance. The flow rate of the infusion pump was adjusted to maintain a mean arterial pressure (MAP) of 60–70 mmHg. Controlled mechanical ventilation was utilized to maintain a partial pressure of end-tidal carbon dioxide (PetCO_2_) of 35–40 mmHg by alteration of ventilatory frequency and tidal volumes. Depending on the patient’s weight, continuous intravenous infusion of Ringer’s fluid to replenish the physiological fluid requirement. At the end of the surgery, all patients were administered intravenous tropisetron 5 mg, patients were tracheal extubated in the operating theater. The same anesthesia technique was performed by the same team of anesthesiologists.

Patients were positioned supine with the head slightly tilted to the nonoperative side, and the head of bed was elevated at 20°^13^. After lidocaine (2% with 1:100 000 epinephrine) was injected locally, a preauricular or retroauricular approach was used to expose the surface of the mastoid bone. The posterior superior spine of the external auditory canal is identified and the mastoid, tympanic sinus, and tympanic chamber are opened through the mastoid pathway with microscopic assistance. Standard modified radical mastoidectomy was performed by the same team of experienced otolaryngologists.

### Interventions

Before skin incision, patients received intravenous TXA (1 g in 20 ml of normal saline) or 20 ml of normal saline administered for 10 min. The chief anesthesiologist, blinded to the group assignment, will administer the product provided by the pharmacy staff.

### Outcome measures

The primary outcome measure is the surgical field clarity score. In this study, all operations and visual fields under the operating microscope during the entire procedure will be videotaped. Finally, the video will be viewed by two independent and trained investigators not involved in the trial, and the clarity of the surgical field will be evaluated based on the Modena Bleeding Score^14^. After independent rating, blinded to each other’s scores, an average of the two scores was recorded as the final result (Table 1).

**Table 1:** The modena bleeding score.

	Scoring
No bleeding	1
Bleeding easily controlled by suctioning, washing, or packing without any significant modification or slowing of surgical procedure	2
Bleeding slowing surgical procedure	3
Most of the maneuvers dedicated to bleeding control	4
Bleeding that prevents every surgical procedure except those dedicated to bleeding control	5

The secondary outcome measures were operation time and the surgeon satisfaction with the surgical conditions using the 0 (worst) to 10 (best) Numeric Rating Scale at the end of surgery. Laboratory examination including hemoglobin, plate count, partial thromboplastin time (APTT), prothrombin time (PT), fibrinogen, and D-dimer at 24 h postoperatively. In addition, general adverse events including nausea, vomiting, dizziness, and serious adverse events such as arterial and venous thromboembolism, myocardial infarction and seizures will be recorded based on clinical signs or symptoms at postoperative 24 h, 3 months, 6 months, and 12 months follow-up to assess the safety of intravenous TXA.

### Sample size calculation

According to the results of the preliminary pilot study, a total of 20 patients were randomized to the TXA and control groups with an equal ratio of 1:1. The primary outcome of The Modena Bleeding Score for the TXA group and control group were 2.5±0.71 and 3±0.47, respectively. We used STATA 16.0 for the sample size calculation, with a power of 80% and an α-level of 0.05, and considering a 15% drop-out rate, at least 28 patients are needed in each group, for a total of 56 patients.

### Statistical analysis

Intention-to-treat analysis (ITT) was employed for data analysis using SPSS 22.0 (IBM Corporation) statistical software. Continuous variables were represented by mean (±SD) and analyzed using a *t*-test or Mann–Whitney *U*-test, while frequencies (percentages) were summarized for categorical variables and analyzed using a *χ*^2^ test or Fisher’s exact test. Two-way repeated-measures analysis of variance (ANOVA) was performed for repeated measures. A *P*-value of <0.05 was considered statistically significant for all experimental variables.

## Results

**Figure 1:**
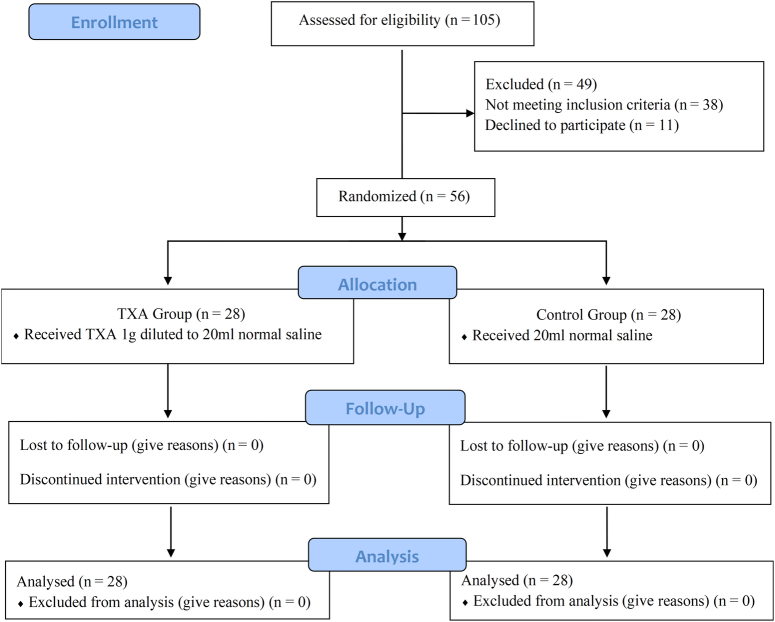
The flow chart of the study.

**Figure 2:**
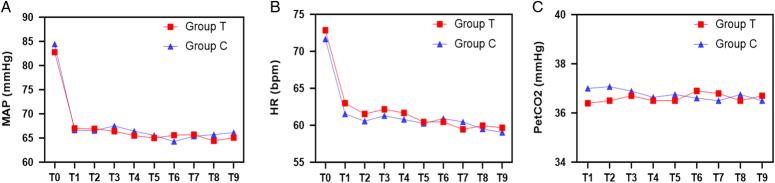
Comparison of intraoperative blood pressure (A), heart rate (B), and PetCO_2_ (C).

From December 2021 to December 2022, a total of 105 patients with chronic suppurative otitis media required microscopic modified radical mastoidectomy were enrolled. Among them, 38 did not meet all inclusion criteria or met exclusion criteria and 11 declined to participate. The final 56 patients (28 TXA group and 28 control group) were randomized and completed the surgery and subsequent follow-up (Fig. 1). All participants were included in the ITT analysis. The baseline demographic characteristics and coagulation profile of the two groups were compared and no statistically significant differences were found, four and three patients performed a second surgery in the TXA group and control group, respectively (Table 2). Figure 2 (A, B, C) showed no differences between groups regarding trends in intraoperative blood pressure, heart rate, and PetCO_2_, respectively (*P*>0.05).

**Table 2:** Demographic data and preoperative laboratory parameters in the two groups.

Variables	TXA group (*n*=28)	Control group (*n*=28)	*P*
Age (mean±SD, years)	44.96±12.62	46.21±11.52	0.453
Sex (*n*, %)			0.280
Male	14 (50.0%)	10 (35.71%)	
Female	14 (50.0%)	18 (64.29%)	
ASA (*n*, %)			0.788
I	13 (46.43%)	12 (42.86%)	
II	15 (53.57%)	16 (57.14%)	
Comorbidities (*n*, %)			
Diabetes	3	4	0.686
Hypothyroidism	2	1	0.553
Asthma	1	0	0.313
BMI (mean±SD, kg/m^2^)	24.42±2.76	24.80±2.59	0.553
SBP (mean±SD, mmHg)	123.21±8.72	120.82±8.47	0.302
DBP (mean±SD, mmHg)	71.93±7.97	69.79±5.29	0.242
Second surgery (*n*, %)	3 (10.71%)	4 (14.29%)	0.686
Hb (mean±SD, g/l)	127.96±7.17	130.71±9.93	0.240
PLT (mean±SD,×10^9)	236.39±36.36	239.71±45.91	0.765
PT (mean±SD, s)	11.44±0.72	11.46±0.68	0.909
APTT (mean±SD, s)	32.24±2.57	31.76±2.01	0.444
D-D (mean±SD)	70.36±29.12	67.93±33.62	0.774
FIB (mean±SD, mg/dl)	270.71±44.84	281.50±43.80	0.367

Data are presented as means±SD/*n*, percentage.

ASA, American Society of Anesthesiologists; DBP, diastolic blood pressure; D-D, D dimer; FIB, fibrinogen; Hb, hemoglobin; PLT, platelet; PT, prothrombin time; PTT, partial thromboplastin time; SBP, systolic blood pressure.

**Figure 3:**
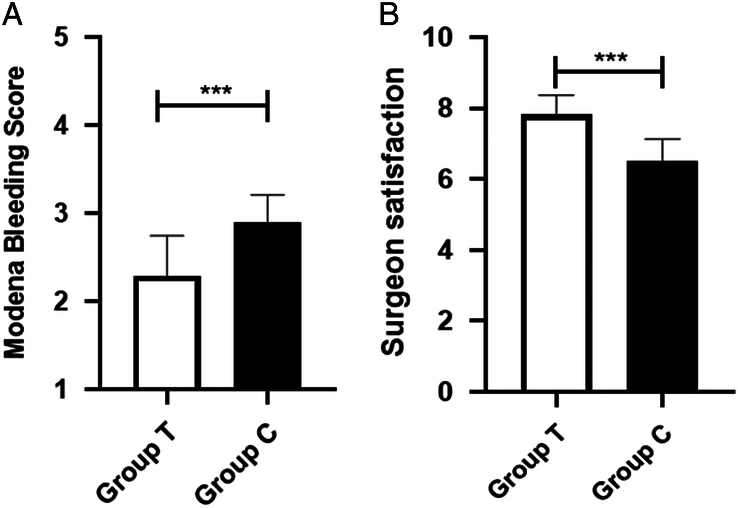
Modena Bleeding Score (A) and surgeon satisfaction in surgical field (B).

The surgical field clarity was significantly lower among the TXA group compared with the control group (2.29±0.46 vs. 2.89±0.31, *P*<0.001) (Fig. 3A). In terms of operation time, the TXA group was significantly shorter than that of the control group (88.61±10.9 vs. 105.2±15.9, *P*<0.001). Accordingly, surgeons in the TXA group were more satisfied with the surgical field than the control group (7.82±0.55 vs. 6.50±0.64, *P*<0.001) (Fig. 3B). Postoperative coagulation parameters were similar between the two groups, and there were three patients in the TXA group and two in the control group who developed mild postoperative nausea (*P*=0.639) (Table 3).

**Table 3:** Outcome measures and postoperative laboratory parameters in the two groups.

Outcome	TXA group (*n*=28)	Placebo group (*n*=28)	*P*
Operation time (mean±SD, min)	88.61±10.89	105.20±15.86	*P*<0.001
Surgical field scores (mean±SD)	2.29±0.46	2.89±0.31	*P*<0.001
Surgeon satisfaction (mean±SD)	7.82±0.55	6.50±0.64	*P*<0.001
Nausea (*n*, %)	3 (10.71%)	2 (7.14%)	0.639
Fluid infusion (mean±SD, ml)	764.29±91.14	807.14±85.76	0.076
Hb (mean±SD, g/l)	129.43±5.63	132.14±5.23	0.067
PLT (mean±SD,×10^9)	232.28±45.41	238.50±46.59	0.609
PT (mean±SD, s)	11.35±0.81	11.45±0.75	0.609
APTT (mean±SD, s)	32.60±3.38	31.90±2.18	0.356
D-D (mean±SD)	84.39±27.33	74.46±15.82	0.102
FIB (mean±SD, mg/dl)	260.32±36.51	276.89±37.04	0.098

Data are presented as means±SD/*n*, percentage.

It is worth mentioning here no participants in either group had a thromboembolic or seizure event during a follow-up period of 12 months.

## Discussion

This study employed rigorous methodology by implementing standard random assignment and blindness, as well as strict inclusion and exclusion criteria. To minimize potential bias, the perioperative management was carried out by the same group of otologists and anesthesiologists. Our findings indicated that in addition to the implementation of other standard measures to decrease intraoperative bleeding, the intravenous administration of 1 g TXA can further enhance the visualization of the surgical field during microscopic middle ear surgery, shorten the operation time, and increase the surgeon satisfaction without significant adverse events.

In microscopic modified radical surgery, the quality of the surgical field of view and visual effects are considered crucial factors. These factors are closely associated with intraoperative bleeding. However, the amount of blood lost during this procedure is generally small and difficult to measure accurately, and measuring total bleeding after surgery does not provide a dynamic assessment of bleeding during the procedure. Therefore, this study used the clarity of the surgical field as the primary outcome of our study. The study recorded the entire operation under the microscope, and two researchers watched the video and scored the clarity of visual field independently according to the Modena Bleeding Score, which could better represent the clarity of the visual field during the entire operation. The Modena Bleeding Score allows for dynamic assessment of bleeding during surgery and has been shown to be an effective method for assessing bleeding in ear surgery. Furthermore, the Modena Bleeding Score is easy to understand and demonstrates excellent interobserver and intraobserver reliability among both individual raters and different raters^15^. Operation time and surgeon satisfaction can serve as indicators of the quality of surgical operating conditions. In our study, patients in the TXA group experienced shorter operation times and higher surgeon satisfaction, which indirectly suggests that the use of TXA can enhance the clarity of the operating field.

Previous studies have demonstrated that a preoperative loading dose of 1 g of TXA can maintain a blood concentration greater than 10 ug/ml in vivo for up to 3 h. Additionally, a pharmacokinetic study involving three healthy volunteers reported that the minimum effective therapeutic plasma concentration of TXA ranged from 5 to 10 ug/ml^16^. Considering that modified radical mastoidectomy procedures typically involve less bleeding and have a shorter operation time of around 2 h, and previous clinical studies have indicated that there is no additional benefit in terms of efficacy when using continuous infusion of TXA for primary hip arthroplasty^17^. Taking these above into account, this study opted for the simple and convenient administration route of a single bolus intravenous injection of TXA.

Due to its antifibrinolytic properties, clinicians have always been concerned about the potential risk of inducing thrombosis with TXA. However, current meta-analysis results have shown that TXA does not increase the risk of thrombotic events or seizures^18^. While there have been isolated case reports of serious adverse events, these have mostly occurred in patients with pre-existing conditions or who received much higher doses of TXA through continuous infusion over a longer period^5^. Laboratory tests conducted in our study also revealed no changes in coagulation indicators at postoperative 24 h, to prove that theoretically, it would not affect the occurrence of thrombosis events. Furthermore, no serious adverse events have been reported in clinical studies using the same treatment regimen^19,20^.

TXA has been proven to be an effective antifibrinolytic drug for reducing perioperative bleeding, demonstrating high cost-effectiveness^21^. Its application effectively reduces intraoperative bleeding and the need for blood transfusion, resulting in savings of blood products and a decrease in transfusion-related injuries^22,23^. However, limited attention has been given to studying its effectiveness in surgeries with minimal bleeding and a high requirement for visual clarity. The findings of this study confirm that TXA significantly improves the clarity of the surgical visual field and reduces operation time, which improves operating room utilization efficiency. The result is consistent with Coombs *et al*.^24^ research on TXA in cosmetic surgery.

There are several strengths to our study. Firstly, this study design adhered to a strict randomized, controlled, and blind method. The demographic characteristics and laboratory results of both groups were similar, and the anesthesia and surgery for all patients included in the study were performed by the same group of otologists and anesthesiologists, effectively eliminating potential bias. Secondly, the entire procedure was videotaped, and two independent researchers evaluated it based on a single overall assessment, which better reflects the clarity of the surgical field throughout the procedure. Thirdly, the patients were followed for 12 months, a sufficient duration to possibly demonstrate the safety of the TXA administration regimen. However, the study also had a few limitations. Firstly, the sample size was small and calculated based on the surgical visual field clarity score, which may limit its statistical validity for evaluating other outcome measures. Secondly, the assessment of thrombotic events relied on clinical signs and symptoms rather than ultrasonography, which could be less accurate.

In conclusion, the study demonstrated that the intravenous administration of a single bolus of 1 g TXA can enhance surgical visualization and reduce operation time without any adverse events associated with TXA, even when all other factors are implemented to provide a clear surgical field. Therefore, provided that attention is given to contraindications, we cautiously recommend the use of TXA as an adjunctive measure during microscopic middle ear surgery. Further large-scale clinical trials are needed to investigate the optimal dosage and administration route further, as well as to expand its indications.

## Ethical approval

Ethical approval was given by the Peking University People’s Hospital Research Ethics Committee (2021PHB173-001).

## Consent

Written informed consent was obtained from the patient for publication of this study and accompanying images. A copy of the written consent is available for review by the Editor-in-Chief of this journal on request.

## Sources of funding

This work did not have any foundation support.

## Author contribution

Y.P.Z. and L.H.: collecting the data, formal analysis, writing – original draft, and visualization; W.S.D. and L.G.: data curation and investigation; Y.F. and H.Y.A: study concept and design, and writing – review and editing.

## Conflicts of interest disclosure

The authors declare that they have no conflicts of interest.

## Research registration unique identifying number (UIN)

This study was registered on the Chinese Clinical Trial Registry (ChiCTR2100049183).

## Guarantor

H.Y. An.

## Data availability statement

The original data will be made available upon reasonable request to the corresponding authors.

## References

[1] Deepika V Ahuja V Thapa D . Evaluation of analgesic efficacy of superficial cervical plexus block in patients undergoing modified radical mastoidectomy: a randomised controlled trial. Indian J Anaesth 2021;65(Suppl 3):S115–s20. 34703056 10.4103/ija.ija_339_21 PMC8500200

[2] Lourijsen E Avdeeva K Gan KL . Tranexamic acid for the reduction of bleeding during functional endoscopic sinus surgery. Cochrane Database Syst Rev 2023;2:Cd012843. 36808096 10.1002/14651858.CD012843.pub2 PMC9943060

[3] El-Ozairy HSE Mady OM Tawfik GM . Outcomes of combined use of topical and intravenous tranexamic acid on surgical field quality during functional endoscopic sinus surgery: randomized controlled trial. Head Neck 2021;43:1389–1397. 33522019 10.1002/hed.26610

[4] Ker K Edwards P Perel P . Effect of tranexamic acid on surgical bleeding: systematic review and cumulative meta-analysis. BMJ (Clinical research ed) 2012;344:e3054. 10.1136/bmj.e3054 PMC3356857 22611164

[5] Dunn CJ Goa KL . Tranexamic acid: a review of its use in surgery and other indications. Drugs 1999;57:1005–1032. 10400410 10.2165/00003495-199957060-00017

[6] Dixon AL McCully BH Rick EA . Tranexamic acid administration in the field does not affect admission thromboelastography after traumatic brain injury. J Trauma Acute Care Surg 2020;89:900–907. 33105308 10.1097/TA.0000000000002932 PMC7878849

[7] Taeuber I Weibel S Herrmann E . Association of intravenous tranexamic acid with thromboembolic events and mortality: a systematic review, meta-analysis, and meta-regression. JAMA Surg 2021;156:e210884. 33851983 10.1001/jamasurg.2021.0884 PMC8047805

[8] McCormack PL . Tranexamic acid: a review of its use in the treatment of hyperfibrinolysis. Drugs 2012;72:585–617. 22397329 10.2165/11209070-000000000-00000

[9] Henry DA Carless PA Moxey AJ . Anti-fibrinolytic use for minimising perioperative allogeneic blood transfusion. Cochrane Database Syst Rev 2011;2011:Cd001886. 11279735 10.1002/14651858.CD001886

[10] Franchini M Focosi D Zaffanello M . Efficacy and safety of tranexamic acid in acute haemorrhage. BMJ (Clinical research ed) 2024;384:e075720. 10.1136/bmj-2023-075720 38176733

[11] Schulz KF Altman DG Moher D . CONSORT 2010 statement: updated guidelines for reporting parallel group randomised trials. Int J Surg 2011;9:672–677. 22019563 10.1016/j.ijsu.2011.09.004

[12] He H Chi Y Yang Y . Early individualized positive end-expiratory pressure guided by electrical impedance tomography in acute respiratory distress syndrome: a randomized controlled clinical trial. Crit Care 2021;25:230. 34193224 10.1186/s13054-021-03645-y PMC8243615

[13] Gan EC Habib AR Rajwani A . Five-degree, 10-degree, and 20-degree reverse Trendelenburg position during functional endoscopic sinus surgery: a double-blind randomized controlled trial. Int Forum Allergy Rhinol 2014;4:61–68. 24282136 10.1002/alr.21249

[14] Alicandri-Ciufelli M Pingani L Mariano D . Rating surgical field quality in endoscopic ear surgery: proposal and validation of the Modena Bleeding Score. Eur Arch Otorhinolaryngol 2019;276:383–388. 30604058 10.1007/s00405-018-05268-6

[15] Athanasiadis T Beule A Embate J . Standardized video-endoscopy and surgical field grading scale for endoscopic sinus surgery: a multi-centre study. Laryngoscope 2008;118:314–319. 17989575 10.1097/MLG.0b013e318157f764

[16] Bolam SM O’Regan-Brown A Konar S . Cytotoxicity of tranexamic acid to tendon and bone in vitro: is there a safe dosage? J Orthop Surg 2022;17:273. 10.1186/s13018-022-03167-5 PMC9107642 35570313

[17] Yang YZ Cheng QH Zhang AR . Efficacy and safety of single- and double-dose intravenous tranexamic acid in hip and knee arthroplasty: a systematic review and meta-analysis. J Orthop Surg 2023;18:593. 10.1186/s13018-023-03929-9 PMC10413625 37563702

[18] Murao S Nakata H Roberts I . Effect of tranexamic acid on thrombotic events and seizures in bleeding patients: a systematic review and meta-analysis. Crit Care 2021;25:380. 34724964 10.1186/s13054-021-03799-9 PMC8561958

[19] Sershon RA Fillingham YA Abdel MP . The optimal dosing regimen for tranexamic acid in revision total hip arthroplasty: a multicenter randomized clinical trial. J Bone Joint Surg Am 2020;102:1883–1890. 33148955 10.2106/JBJS.20.00010

[20] Devereaux PJ Marcucci M Painter TW . Tranexamic acid in patients undergoing noncardiac surgery. N Engl J Med 2022;386:1986–1997. 35363452 10.1056/NEJMoa2201171

[21] Karl V Thorn S Mathes T . Association of tranexamic acid administration with mortality and thromboembolic events in patients with traumatic injury: a systematic review and meta-analysis. JAMA Network Open 2022;5:e220625. 35230436 10.1001/jamanetworkopen.2022.0625 PMC8889461

[22] Gayet-Ageron A Prieto-Merino D Ker K . Effect of treatment delay on the effectiveness and safety of antifibrinolytics in acute severe haemorrhage: a meta-analysis of individual patient-level data from 40 138 bleeding patients. Lancet (London, England) 2018;391:125–132. 29126600 10.1016/S0140-6736(17)32455-8 PMC5773762

[23] Hunt BJ . The current place of tranexamic acid in the management of bleeding. Anaesthesia 2015;70(Suppl 1):50–3, e18. 25440395 10.1111/anae.12910

[24] Coombs DM Kwiecien GJ Sinclair NR . Local infiltration of tranexamic acid during facelift improves operating room efficiency: a matched patient study. Aesthet Surg J 2022;42:971–977. 35350068 10.1093/asj/sjac067

